# Zeolite Cotton in Tube: A Simple Robust Household Water Treatment Filter for Heavy Metal Removal

**DOI:** 10.1038/s41598-020-61776-8

**Published:** 2020-03-13

**Authors:** Xutao Chen, Lisha Yu, Shihui Zou, Liping Xiao, Jie Fan

**Affiliations:** Key Lab of Applied Chemistry of Zhejiang Province, Department of Chemistry, Zhejiang University, Hangzhou, 310027 China

**Keywords:** Environmental chemistry, Materials science

## Abstract

It is challenging to develop a low-cost household water treatment (HWT) that simultaneously deliver an effective and robust way for safe and reliable water supply. Here, we report a simple flow-through filter made by zeolite-cotton packing in a tube (ZCT) as low-cost HWT device to remove heavy metal ions from contaminated water. The zeolite-cotton is fabricated by an on-site template-free growth route that tightly binds mesoporous single-crystal chabazite zeolite onto the surface of cotton fibers. As a result, the ZCT set-up with optimized diameter achieves both high adsorption efficiency, proper flow rate, reliable supply and strong stability at the same time. After flowed through the set up packed with 10 g of zeolite-cotton, 65 mL 1000 ppm Cu^2+^ solution was purified down to its safety limit (<1 ppm). Notably, their efficiency remains unaltered when filtering several ions simultaneously. In a simulated purification process, 8 L of water contaminated by Cu^2+^, Cd^2+^ and Pb^2+^ could be transformed into drinking water and it enables the removal of heavy metals to concentrations of below 5 ppb (μg L^−1^). We also show that the ZCT can be used for disinfection by introducing Ag-exchanged zeolite-cotton without contaminating the water with Ag ions (<0.05 ppm).

## Introduction

Drinking water safety is one of the most serious health problems throughout the world, especially for those people in relatively undeveloped districts. Health risks may arise from consumption of water contaminated with numerous pollutants such as heavy metal ions, persistent organic pollutants, pharmaceutical waste, virus and microbial pathegens^1^. As reported by World Health Organization (WHO), nearly 2 billion people are using either un-improved drinking water source or faecally-contaminated water. Close to half a million diarrheal deaths in low- and middle-income countries are attributed to unsafe drinking-water, and the vast majority of these deaths occur among children under five^2^. Household water treatment (HWT) is an important public health intervention to improve the quality of drinking-water, particularly among those who rely on water from unimproved sources. Further, safe drinking-water is an immediate priority in most emergencies, and HWT can be an effective emergency response intervention^3,4^, for example, the project solar disinfection (SODIS) of drinking water^5–8^, which is awarded the 2020 UNESCO Prizes, is an important HWT technique approved by WHO.

Unlike organic pollutants, heavy metal ions in water is hard to notice and avoid but pose a long-term threat to human. Heavy metal contaminated water usually has no visible difference as compared to safe drinking water, however, every little intake will not degrade but accumulate in human body. Even if the heavy metal-polluted water is not directly taken by human, it can also accumulate in algae, shellfish and fish, then concentrate through food chain and finally result in serious disease such as encephalopathy, hemolytic anemia or even cancer^9–11^. Those global issues have reminded people to search for more proper ways to de-contaminate water against heavy metal ions.

Cost is priority for undeveloped districts^12^. Traditional water treatment methods include ion exchange resin/zeolite^13–16^, chemical precipitation^17,18^, electro-chemical treatment^19–21^ and reverse osmosis^22,23^, *et al*. The latter three treatment methods are often chemically, energetically and operationally intensive, focused on large systems, and thus require considerable infusion of capital, engineering expertise and infrastructure^24^, all of which precludes their use in much of the world. The cost of ion-exchange resins or zeolite is only about 1/3~1/10 of other metioned treatments^25^, making it the most economic technique to remove heavy metal ions and other pollutants as HWT. However, due to slow ion diffusion, it generally suffers from low efficiencies (60–90%)^26,27^ which needs a large mass loading to satisfy high treatment rate demands^28^. It is of great importance to develop an effective, lower-cost, robust methods to decontaminate waters from source to point-of-use, without further stressing the environment or endangering human health by the treatment itself.

Herein, we develop a simple HWT filter device that is made by zeolite cotton packed in a tube (ZCT). Zeolite cotton (ZC) is prepared by growth of mesoporous zeolite CHA (mCHA) onto the surface of cotton fiber. ZC hybrid material exhibits superior ion-exchange activity and outperforms the conventionally zeolite granules or zeolite powder impregnated cotton cloth in terms of high heavy ion removal capacity and short flow-through time, easy operation and low cost. It achieved not only an ionic adsorption capacity for over 6.5 mg g^−1^ (Cu^2+^) but also a treatment efficiency of 84 mL day^−1^ g^−1^, enabling the removal of heavy metals to the concentrations below 5 ppb (μg L^−1^). The performance of the flow-through filter is enabled by the ability of the zeolite nanocrystals to selectively absorb heavy metal pollutants from solutions. We also showed that the ZCT can be used for disinfection by introducing Ag-exchanged ZC without contaminating the water with excessive Ag ions (<0.05 ppm). These performance parameters demonstrate that this simple flow-through filter not only has high efficiency, capacity, and water cleaning power, but also requires no energy input and is easy to set up. We envision that ZCT could be an economically viable process for a daily drinking water purification for undeveloped regions.

## Results and discussion

### Characterization of ZC

**Figure 1:**
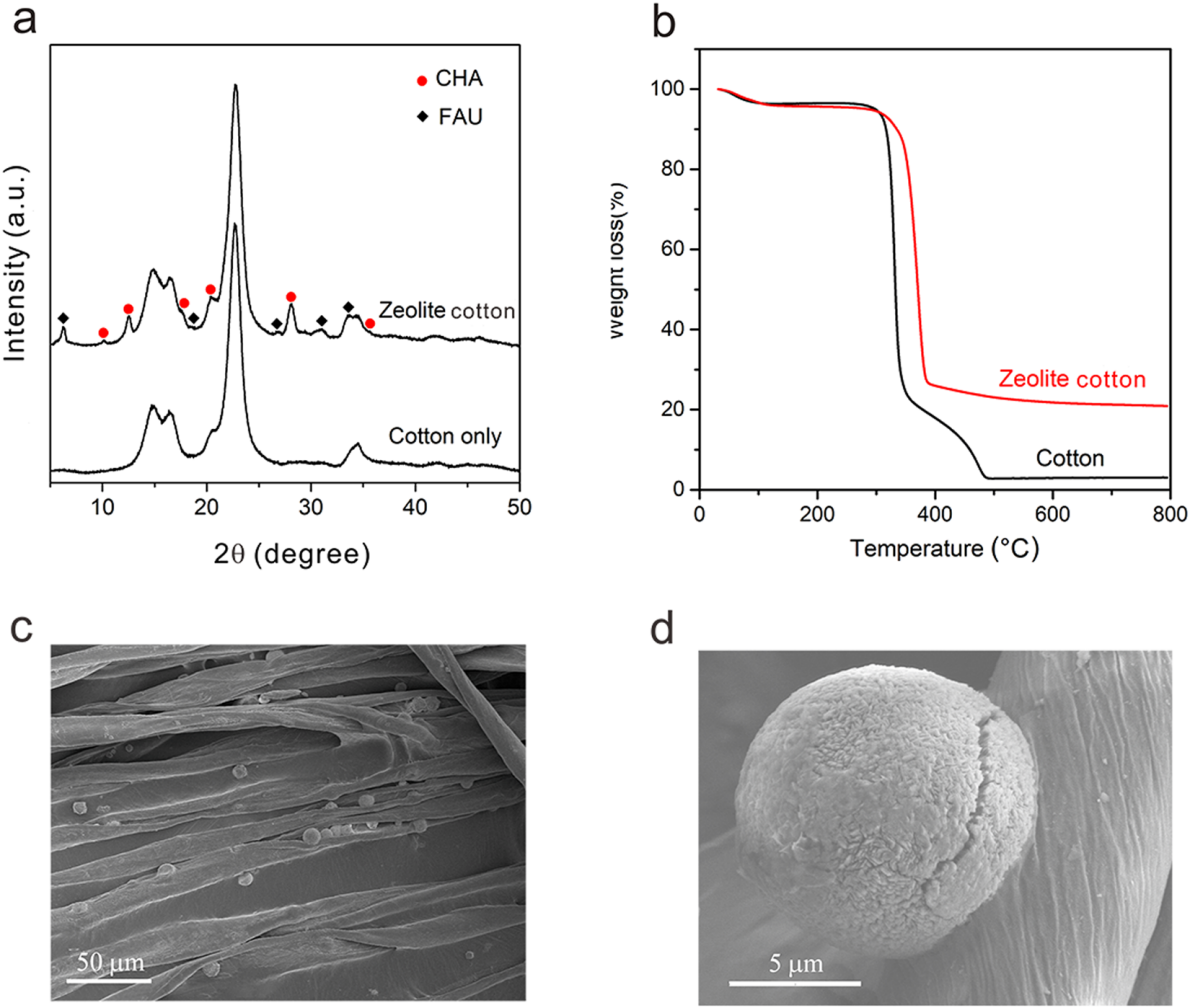
Characterization of ZC (**a**) XRD patterns. (**b**) TG analysis of ZC and cotton only. (**c**,**d**) SEM images of ZC, hemi-spherical like particle is CHA and plate like particle is FAU, indicating that majority of zeolite is indexed to CHA.

Figure 1a shows the X-ray diffraction (XRD) patterns of ZC. The peaks appearing at 12.56°, 17.64°, 20.44°, 28.12° and 35.56° can be readily indexed as chabazite (JCPDS: 34–0137) while the peaks located at 6.27°, 10.14°, 18.62°, 26.86°, 31.05° and 33.64° matches with the standard faujasite pattern (JCPDS: 12–0228). These two kinds of zeolite are also observed by Scanning Election Microscope (SEM). As shown in Fig. 1c, the majority of the zeolites have hemi-spherical morphology, corresponding to CHA, in good line with our previous report^29^. The plate-like particles, on the other hand, are FAU. Particle size of CHA and FAU are both around 10 μm. Interestingly, the spherical-like CHA particles are tightly bonded with rod-like cotton fibers, giving a strong connection between the zeolite and cotton, which makes the zeolite hard to fall off from the fiber (Fig. 1d). Thermal gravity analysis (TGA) result reveals that the average zeolite loading of ZC is 20.27 ± 0.7w% (Fig. 1b). Chemical formulation of the zeolite is determined by XRF to be (Na_2_O)_0.43_(SiO_2_)_0.90_(Al_2_O_3_)_0.24_.

### ZCT set up

**Figure 2:**
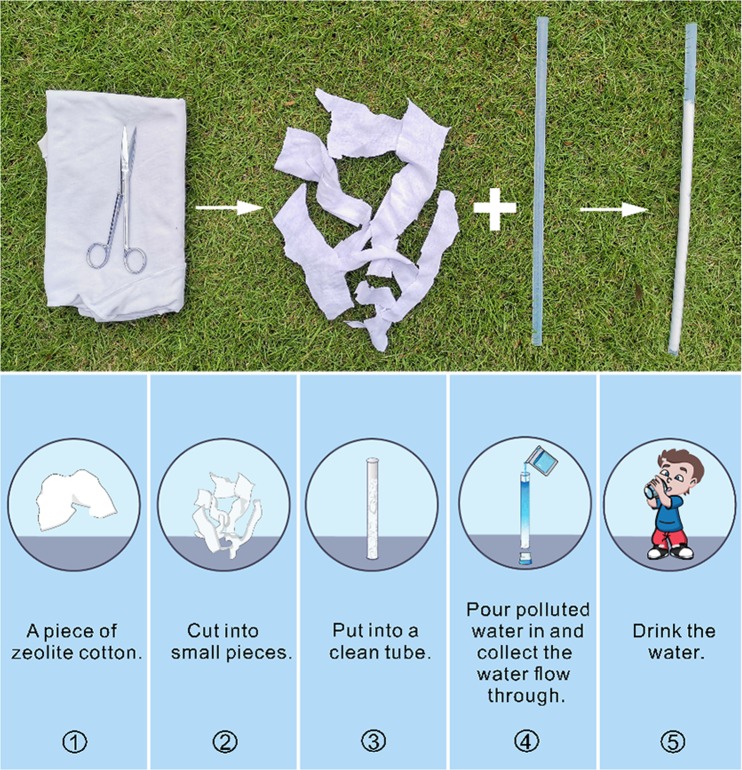
The set up of HWT filter ZCT and its application procedure to collect drinking water.

The procedure to set up ZCT is very simple. All steps are shown in Fig. 2. A piece of zeolite cotton was cut into small pieces and then put into a clean tube. Polluted water can be then poured into the tube and after several tens of minutes, the water flowed through would be transformed into qualified drinking water. No extra energy input, operating skills, spaces or complicate facilities are needed. Everyone can set up this kind of HWT at his/her home using any kind of tubes, bottles or cans available to him/her.

### Heavy metal removal

**Figure 3:**
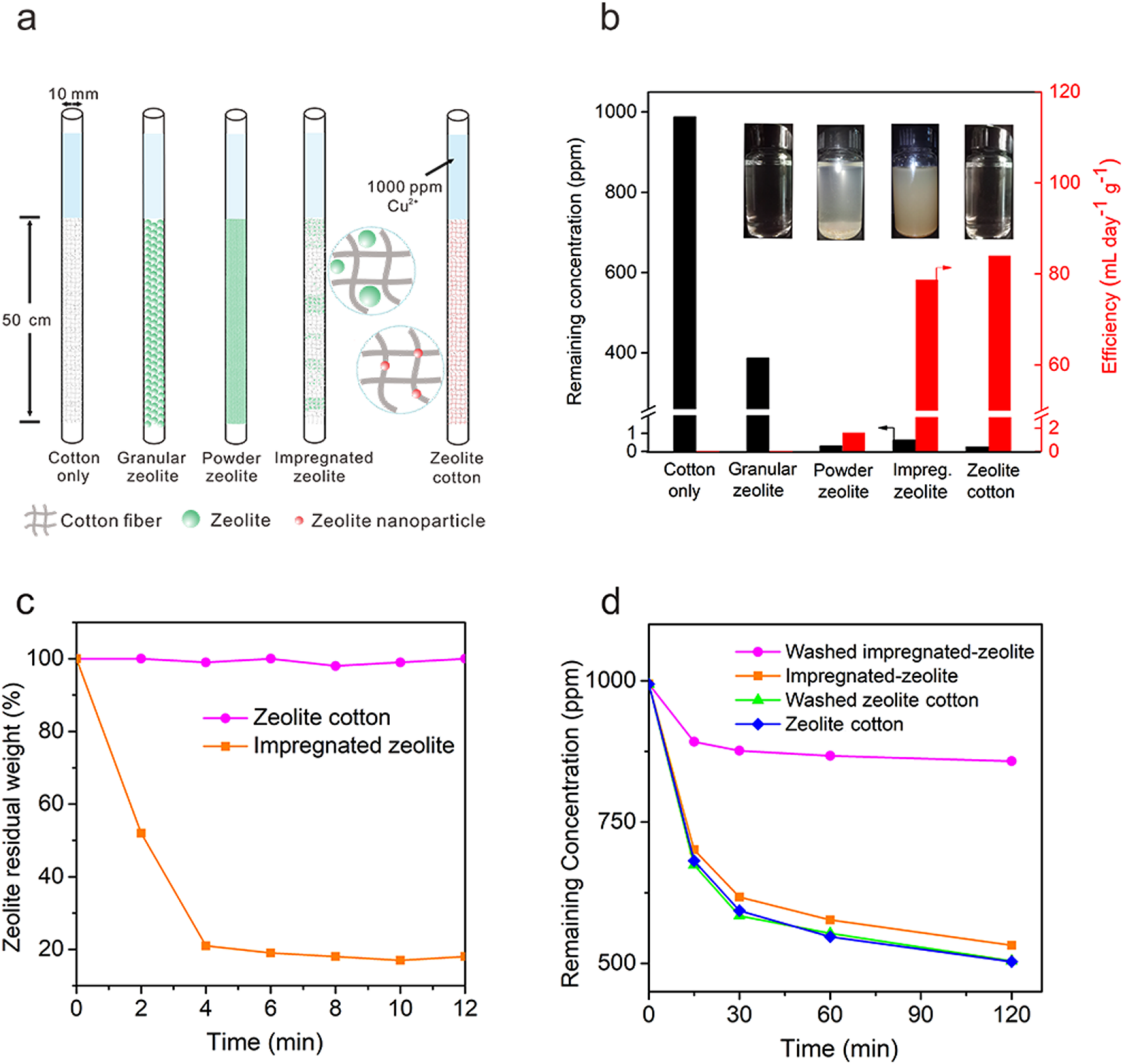
Comparison of granular zeolite, powder zeolite, impregnated zeolite and ZC. (**a**) Schematic of these four kinds of zeolite packing in the tube. (**b**) Cu^2+^ remaining concentration and removal efficiency of four zeolite and the optical images of the collected water (inset). (**c**) Residual weight of zeolite on impregnated zeolite and ZC after ultrasonic clean. (**d**) Cu^2+^ static adsorption performance of impregnated zeolite and ZC before and after hand-washing.

A series of experiments were conducted to evaluate the heavy metal removal properties (Cu^2+^ as an example) of ZC and traditional zeolite. As suggested by WHO, the safety limit for Cu^2+^ in drinking water is 1 ppm. Here, the filtration system is shown in Fig. 3a. Four types of zeolite: granular zeolite, powder zeolite, impregnated zeolite and ZC were added into four plastic tubes (d = 10 mm) under the same filling height (50 cm). Pure cotton was set as a reference. The mass of zeolite was determined to be 20.9 g, 32.4 g, 2.0 g and 2.0 for granular zeolite, powder zeolite, impregnated zeolite and ZC, respectively. The treatment efficiency is defined as the volume of safe water (Cu^2+^ <1 ppm) purified by every 1 gram of material per day. As shown in Fig. 3b, the Cu^2+^ concentration after filtration was 386.1 ppm for granular zeolite, which is significantly higher than the safety limit. This poor adsorption performance was caused by the slow ionic diffusion rate among the large-particle zeolite and heavy metal ions. The water purified by powder zeolite was below Cu^2+^ safety limit but the flow rate was highly restricted by the dense stacking of zeolite powder so the treatment efficiency was only 1.6 mL day^−1^ g^−1^. In addition, large amount of powder zeolite consumed in this section is another reason for this low treatment efficiency whose unit is mL day^−1^ g^−1^. Impregnated zeolite has both safe water and optimal flow rate. Overall treatment efficiency of impregnated zeolite was 78.3 mL day^−1^ g^−1^. However, the impregnated zeolite has poor connection with cotton so considerable muddiness was observed in the water after purification (Fig. 3b, inset). Only ZC achieved qualified drinking water, high flow rate and strong stability at the same time. The Cu^2+^ concentration after filtration was only 0.3 ppm and the treatment efficiency was 84 mL day^−1^ g^−1^. Finally, 65 mL qualified water was collected before reaching a maximum capacity.

To further compare the stability of ZC and impregnated zeolite, ultrasonic clean was applied and the weight loss percentage of the zeolite was calculated. As shown in Fig. 3c, impregnated zeolite lost around 80% of total zeolite after 4 min ultrasonic clean while no weight loss was observed for the ZC after 10 min, corresponding to different binding strength between zeolite and cotton fiber. Besides, we further compared the static adsorption performance of ZC or impregnated zeolite before and after hand-washing. 1 g of ZC or impregnated zeolite was put into a beaker containing 20 mL of 1000 ppm CuCl_2_ solution, the change of concentration of Cu^2+^ was monitored during the purification process. As shown in Fig. 3d, for the impregnated zeolite sample, there is a significant change of the adsorption curve. The final concentration of Cu^2+^ increased from 532.1 ppm to 857.7 ppm, indicating a 70% loss of its original adsorption capability. On the other hand, ZC adsorption performance stayed unchanged before and after washing. These results clearly demonstrate the superior reliability of ZC over impregnated zeolite.

To summarize, ZC overcomes the drawbacks of granular zeolite and powder zeolite: granular zeolite usually gives very poor heavy metal adsorption performance due to its slow ionic diffusion rate and powder zeolite makes the water hard to flow through. ZC achieved a good adsorption performance and appropriate flow rate simultaneously. In addition, the strong binding of zeolite nanocrystals to the cotton fiber of ZC ensure its safe application during the water treatment, which excludes the re-contaminating water by leached zeolite powders observed in impregnated sample.

**Figure 4:**
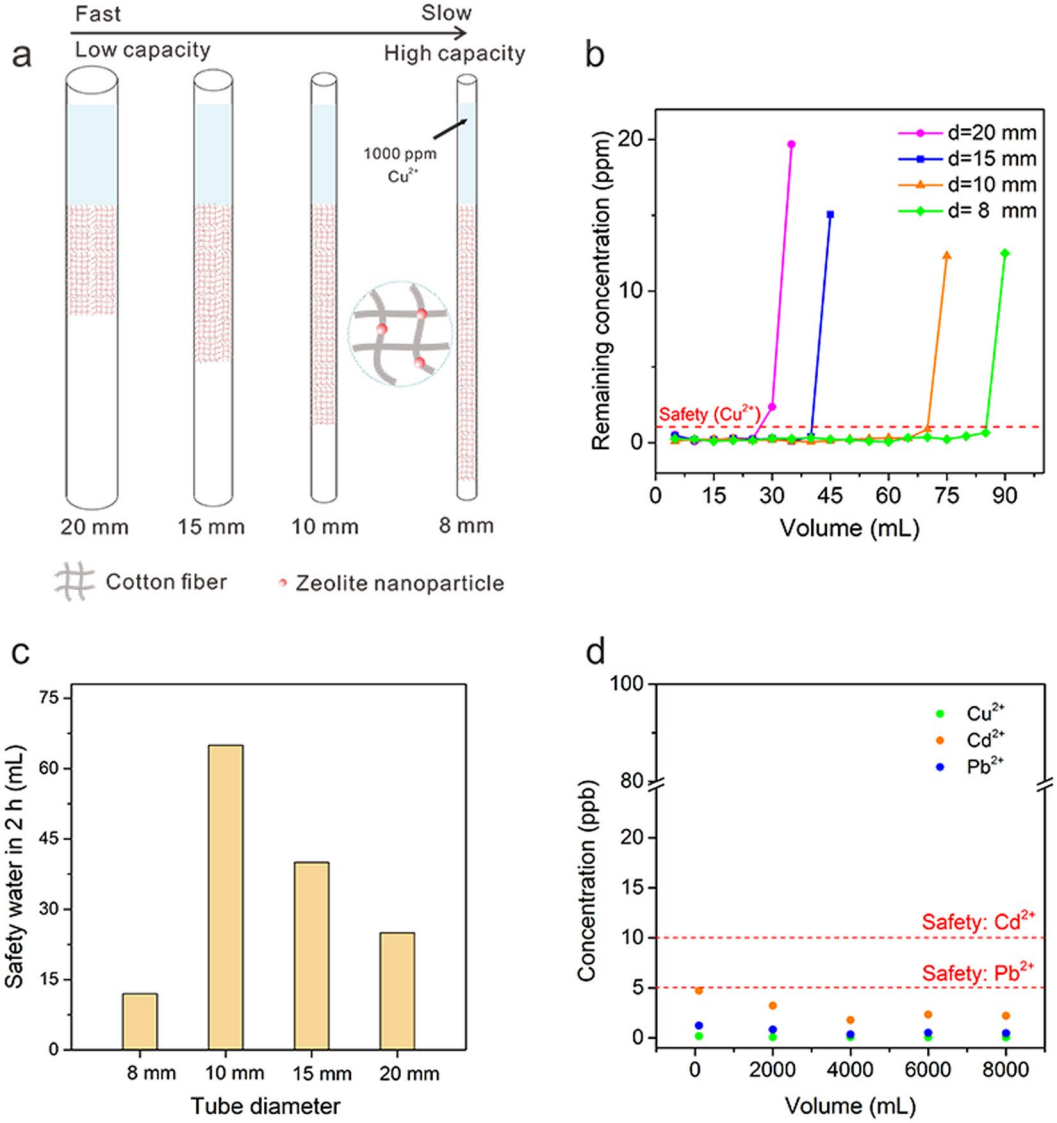
Performance of four ZCT with different tube diameters (20 mm, 15 mm, 10 mm and 5 mm). (**a**) Schematic of 10 g ZC packing in those four tubes. (**b**) Volume of safety water obtained by four tubes, starting concentration: Cu^2+^ = 1000 ppm. (**c**) The volume of the safety water purified by four tubes within 2 h. (**d**) Remaining concentration for long-term flowing with three ions (Cu^2+^, Cd^2+^ and Pb^2+^) with a starting concentration: Cu^2+^ = 4.29 ppm, Cd^2+^ = 3.12 ppm and Pb^2+^ = 14.19 ppm. The flowed through water is below the safety limit for drinking water.

The tube diameter is a key parameter for this HWT filter device. At a given mass of ZC, the tube diameter determines the height of filter and therefore the pathway of the solution. In this study, four tubes with different diameter (20 mm, 15 mm, 10 mm and 8 mm, Fig. 4a) were evaluated. The Cu^2+^ concentration of the initial solution was 1000 ppm. According to Fig. 4b, a maximum of 25, 35, 65 and 85 ml qualified water could be obtained by 20 mm, 15 mm, 10 mm and 8 mm tubes, respectively. Thinner tubes turned more contaminated water into drinking water but the flow rate was slower as well. Increasing tube diameter leads to an obvious decrease of adsorption capacity but the flow rate is significantly enhanced. The flow rate is as low as 6 mL h^−1^ for 8 mm tube, which is too slow for daily use. 20 mm tube has the highest flow rate but its capacity is limited. Among these four tubes, 10 mm tube is the optimal one as it produces the most safety drinking water within 2 h (65 mL, Fig. 4c). Therefore, we selected the 10 mm diameter tube for further experiments.

A real contaminated water purification process needs to remove various kinds of heavy metal ions at the same time. Here, Cu^2+^, Cd^2+^ and Pb^2+^ containing water was used to simulate the real contaminated water. Numerous studies have been reported to remove these three metal ions in water^9,30,31^. Cu^2+^, Cd^2+^ and Pb^2+^ are commonly presented in industrial-polluted waters and all pose various threats to human body. Cd^2+^ accumulation results in lung cancer, proteinuria and osteomalacia after long-term absorption^28,32^. Excessive Pb^2+^ intake causes encephalopathy, inanemia, and nephropathy^33,34^. The toxicity of Cu^2+^ is not as severe as Cd^2+^ and Pb^2+^ but exceeding safe limits of copper can also induce hemolytic anemia and necrotizing hepatitis^28,35^. As suggested by WHO, the upper limit for Cu^2+^, Cd^2+^ and Pb^2+^ in drinking water is 1 ppm, 0.005 ppm and 0.010 ppm, respectively. After literature review of the current levels of heavy metal pollution in Africa^14,36–40^, the initial concentration of Cu^2+^, Cd^2+^ and Pb^2+^ were set to be 4.29 ppm, 3.12 ppm and 14.19 ppm, respectively, similar to that of the water in a south Africa river, reported by Yabe and coworkers^38^. The remaining metal concentration at 2000 mL interval is shown in Fig. 4d. All three metal ions were adsorbed down to their safety limit: Cu^2+^ to ~0.1 ppb, Cd^2+^ to ~3 ppb and Pb^2+^ to ~1 ppb, with consistent values over the entire volume of water. The removal mechanism of heavy metal in water using zeolite is a combination of adsorption and ionic exchange^41,42^. Na^+^ concentration, which is exchanged by Cu^2+^, Cd^2+^ and Pb^2+^, in the flowed through water was around 6~7.5 ppm, fairly less than the WHO suggested limit (200 ppm). Finally, around 8000 mL contaminated water was purified by 10 g ZC. Suppose an adult require 2000 mL drinking water every day and a cloth made of ZC, for example, a T-shirt is 200 g, then the water purified by this T-shirt would be at least 160 L, fairly enough for his/her need for 80 days.

We note that the real wastewater is more complicated than we have tested. Other components including alkali and alkaline earth metal ions available in water might affect the sorption characteristics of the zeolitic materials^43^. Fortunately, the impact of competing cations is considerable only if their concentration is significantly higher than that of heavy metal ions. Zamzow and co-workers^44^ tested the impact of competing cations, including Na^+^ and Ca^2+^, on the uptake of Pb^2+^. They found that the adsorption of 50 ppm of Pb^2+^ did not decrease by Na^+^ and Ca^2+^ until their concentration beyond 1500 ppm. Similar results were also suggested by Panayotova^45^. In addition, from the practicle point of view, it is also possible to increase the flow rate of thinnest tube (8 mm) by connecting to a tap, which allow us to achieve the proper flow rate and satisfied adsorption capacity at the same time.

### Anti-bacterial test

Silver has been well known as an anti-bacterial agent for centuries^46^. After exchanged into the zeolite framework, silver-zeolite also possessed disinfection function. Many researches have explored the anti-bacterial function of silver-zeolite^47–50^. Kwakye-Awuah and co-workers^48^ examined the antimicrobial action and efficacy of 2.0 wt% Ag^+^ loaded zeolite X on *Escherichia coli*, *Staphylococcus aureus* and *Pseudomonas aeruginosa*. They found 1 g/L Ag^+^ loaded zeolite X could purify all three microbial solution from 10^6^ CFU/ml down to 10^1^ CFU/ml within 0.5 h. The silver-loaded zeolite X retained its antimicrobial action after three times of wash and re-try. However, after treated by Ag^+^ loaded zeolite X, the resulted solution contained Ag^+^ ions with a concentration of 1.8–2.5 ppm, which is two-order-of magnitude higher than safety Ag^+^ concentration for drinking water (<0.05 ppm). Excessive intake of Ag^+^ can lead to imbalance of intestinal flora and pose a threat to human health^46^. Therefore, this problem must be solved before applying silver exchanged zeolite into drinking water treatment.

**Figure 5:**
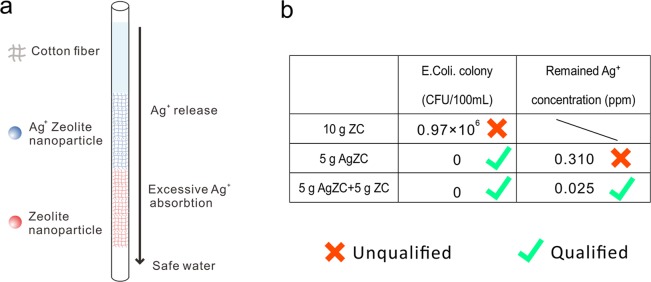
(**a**) The Schematic of dual-bed HWT filter used for anti-bacteria. (**b**) Number of *E. Coli* colony and remained Ag^+^ concentration after filtered through the beds of 10 g ZC, 5 g AgZC and 5 g AgZC + 5 g ZC, respectively.

In this work, we propose a dual-bed filter configuration to solve this problem, in which 5 g of Ag exchanged ZC (AgZC) was set at the top of ZC bed. The mass loading of silver is quite low (1 mg/g) which would not significantly affect the performance of ZC for heavy metal ions. Considering the low cost of silver-exchanged ZC (extra $0.0009/g), it is disposable and does not require a complicated and costly regeneration process. Figure 5a demonstrates the main idea: bacterial-polluted water would be first purified by AgZC then excessive Ag^+^ released from the cotton above would be re-adsorbed by those un-exchanged ZC and finally the water would contain neither bacterial nor overdose Ag^+^. During the experiment, the original bacterial solution had a *Escherichia coli* (*E. coli*) concentration of 0.5~1 × 10^6^ CFU/100 mL. *E. coli* is a traditionally applied indicator to monitor the microbiological water quality since it provide a direct evidence of recent faecal pollution^51^. WHO guideline suggests that no detection of *E. coli* in 100 mL water sample is required to identify a water source safe to drink. As shown in Fig. 5b, the tube packed with 10 g of ZC is unable to kill *E. coli* and final bacterial colony was 0.97 × 10^6^ CFU/100 mL. However, the tube with 5 g of AgZC had an obvious anti-bacterial function. No viable colony was obtained in the flowed through solution but the remained Ag^+^ concentration was 0.310 ppm, 6 times higher than the safety limit. The dual-bed tube packed with 5 g AgZC + 5 g ZC gave a sufficient anti-bacterial activity while Ag^+^ was reduced down to 0.025 ppm which is lower than the safety concentration. Therefore, this kind of device could be used to remove bacterial in water without re-contaminating the water with excess Ag ions, suitable for drinking water.

## Conclusion

Filters composed of cotton and cloths have been used to filter water and other beverages since ancient time, which is only used to reduce water turbidity. In this work, by combining the zeolite together with cotton fibers, we endure the zeolite cotton with the function of heavy metal removal and disinfection: 8 L of simulated waste water could be transformaned into safety drinking water using 10 g ZC packing in a diameter optimized tube. Overall treatment efficiency was 84 mL day^−1^ g^−1^. Silver exchanged ZC was proved to show anti-bacterial property, *E.Colo*. contaminated water could flow through the device with neither remaining *E. Coli* nor excessive Ag^+^. We believe this simple ZCT filter is practical and affordable household water treatment throughout much of the world, and is available for widespread.

## Methods

### Materials

Ludox HS-30 colloidal silica (30 wt% suspension in H_2_O) was purchased from Sigma-Aldrich (Shanghai, China). Sodium hydroxide (NaOH), aluminium hydroxide (Al(OH)_3_), copper chloride (CuCl_2_), cadmium chloride (CdCl_2_), lead nitrate (Pb(NO_3_)_2_) and silver nitrate (AgNO_3_) were obtained from Sinopharm Chemical Reagent Co., Ltd (Shanghai, China). Two complexing agents: ethylenediaminetetraacetic acid (EDTA) and Diethyldithiocarbamic acid sodium salt (DDTC), were purchased from Aladdin (Shanghai, China). The Cotton, which is consisted of 100% cotton cellulose and was pretreated with alkaline boiling, was bought from Xiamen Xinhong Extension Trade Co., Ltd. *E. coli* (CMCC(B)44102), together with R2A medium, were obtained from Shanghai Zhiqiao Biological Engineering Co., Ltd. Commercial zeolite faujausite (CAS: 12173-28-3, (Na_2_O)_43_(SiO_2_)_106_(Al_2_O_3_)_43_) and chabazite (CAS: 12251-32-0, (Na_2_O)_4_(SiO_2_)_16_(Al_2_O_3_)_4_) was purchased from Meryer (Shanghai) Chemical Technology Co., Ltd.

### Synthesis of zeolite cotton

The synthesis procedure is similar to the method reported in literature^29^. A gel precursor with molar ratio 9 Na_2_O:0.7 Al_2_O_3_:10 SiO_2_:331 H_2_O was prepared from solution A and B. Solution A was prepared by dissolving 400 g sodium hydroxide and 109 g aluminum hydroxide into 3200 mL deionized water. Solution B was a mixture from 320 g sodium hydroxide, 1360 mL deionized water and 2000 g colloidal silica (30 wt%). Both A and B was heated until the solution turned clear. A piece of cotton (200 g) was then added into solution B and transferred into ice-water mixture. Solution A was mixed into solution B dropwise with vigorous stirring. The precursor was then placed into a Teflon-lined autoclave and kept at 100 °C for 24 h. The sample was then washed with deionized water for several times and ultrasonic cleaned for 5 min to removal those loosely-bonded zeolite, and finally dried at 65 °C.

### Cu^2+^ filtration comparison

Granular zeolite, powder zeolite, impregnated zeolite and ZC were separately added into four plastic tubes (d = 10 mm) until reaching the same height (h = 50 cm). The weight ratio for granular zeolite, powder zeolite and impregnated zeolite is CHA: FAU = 1:1. CuCl_2_ solution with a Cu^2+^ concentration of 1000 ppm was poured into those tubes and the water flowed down was collected. Remained Cu^2+^ concentration was determined by Ultraviolet-visible Spectrophotometer (UV-Vis) using EDTA and DDTC as complexing agents for high (50–1000 ppm) and low (0.1–50 ppm) concentration, respectively. The absorption wavelength for Cu^2+^-EDTA and Cu^2+^-DDTC is 730 nm and 452 nm, respectively. Handwashing test was conducted by washing 1 g of zeolite cotton or impregnated using 50 mL deionized water for three times. To optimize tube diameter, four tubes with different diameter (20 mm, 15 mm, 10 mm and 8 mm) were tested. In order to simulate the real contaminated waste water, 10 g zeolite-cotton was added into a 10 mm diameter tube, then heavy metal ions solution with a referenced concentration (Cu^2+^ = 4.29 ppm, Cd^2+^ = 3.12 ppm and Pb^2+^ = 14.19 ppm) was poured into the tube. Remained Cu^2+^, Cd^2+^ and Pb^2+^ concentration was characterized by inductively coupled plasma mass spectroscopy (ICP-MS).

### Characterizations

The powder X-ray diffraction (XRD) patterns were recorded on a Rigaku Ultimate IV with Cu Kα radiation (10 °C min^−1^). The accelerating voltage and the applied current were 40 kV and 40 mA, respectively. The morphologies of prepared samples were observed via the field-emission scanning electron microscope (FE-SEM, Hitachi SU8010, Japan). The thermogravimetry analyses (METTLER, TGA/DSC 1/1100, Switzerland) were recorded under dynamic oxygen flow by heating the samples to 800 °C at a rate of 10 °C min^−1^.

## Data Availability

The final dataset and accompanying material are available on request.
